# Machine learning derived ECG risk score improves cardiovascular risk assessment in conjunction with coronary artery calcium scoring

**DOI:** 10.3389/fcvm.2022.976769

**Published:** 2022-10-05

**Authors:** Shruti Siva Kumar, Sadeer Al-Kindi, Nour Tashtish, Varun Rajagopalan, Pingfu Fu, Sanjay Rajagopalan, Anant Madabhushi

**Affiliations:** ^1^Department of Biomedical Engineering, Case Western Reserve University, Cleveland, OH, United States; ^2^Harrington Heart and Vascular Institute, University Hospitals, Cleveland, OH, United States; ^3^School of Medicine, Case Western Reserve University, Cleveland, OH, United States; ^4^Department of Population and Quantitative Health Sciences, Case Western Reserve University, Cleveland, OH, United States; ^5^Wallace H. Coulter Department of Biomedical Engineering, Radiology and Imaging Sciences, Biomedical Informatics (BMI) and Pathology, Georgia Institute of Technology and Emory University, Research Health Scientist, Atlanta Veterans Administration Medical Center, Atlanta, GA, United States

**Keywords:** machine learning, artificial intelligence, atherosclerotic cardiovascular diseases (ASCVD), electrocardiogram (ECG), risk assessment/classification, nomogram

## Abstract

**Background:**

Precision estimation of cardiovascular risk remains the cornerstone of atherosclerotic cardiovascular disease (ASCVD) prevention. While coronary artery calcium (CAC) scoring is the best available non-invasive quantitative modality to evaluate risk of ASCVD, it excludes risk related to prior myocardial infarction, cardiomyopathy, and arrhythmia which are implicated in ASCVD. The high-dimensional and inter-correlated nature of ECG data makes it a good candidate for analysis using machine learning techniques and may provide additional prognostic information not captured by CAC. In this study, we aimed to develop a quantitative ECG risk score (eRiS) to predict major adverse cardiovascular events (MACE) alone, or when added to CAC. Further, we aimed to construct and validate a novel nomogram incorporating ECG, CAC and clinical factors for ASCVD.

**Methods:**

We analyzed 5,864 patients with at least 1 cardiovascular risk factor who underwent CAC scoring and a standard ECG as part of the CLARIFY study (ClinicalTrials.gov Identifier: NCT04075162). Events were defined as myocardial infarction, coronary revascularization, stroke or death. A total of 649 ECG features, consisting of measurements such as amplitude and interval measurements from all deflections in the ECG waveform (53 per lead and 13 overall) were automatically extracted using a clinical software (GE Muse™ Cardiology Information System, GE Healthcare). The data was split into 4 training (S_tr_) and internal validation (S_v_) sets [S_tr_ (1): S_v_ (1): 50:50; S_tr_ (2): S_v_ (2): 60:40; S_tr_ (3): S_v_ (3): 70:30; S_tr_ (4): S_v_ (4): 80:20], and the results were compared across all the subsets. We used the ECG features derived from S_tr_ to develop eRiS. A least absolute shrinkage and selection operator-Cox (LASSO-Cox) regularization model was used for data dimension reduction, feature selection, and eRiS construction. A Cox-proportional hazards model was used to assess the benefit of using an eRiS alone (M_ecg_), CAC alone (M_cac_) and a combination of eRiS and CAC (M_ecg+cac_) for MACE prediction. A nomogram (M_nom_) was further constructed by integrating eRiS with CAC and demographics (age and sex). The primary endpoint of the study was the assessment of the performance of M_ecg_, M_cac_, M_ecg+cac_ and M_nom_ in predicting CV disease-free survival in ASCVD.

**Findings:**

Over a median follow-up of 14 months, 494 patients had MACE. The feature selection strategy preserved only about 18% of the features that were consistent across the various strata (S_tr_). The M_ecg_ model, comprising of eRiS alone was found to be significantly associated with MACE and had good discrimination of MACE (C-Index: 0.7, *p* = <2e-16). eRiS could predict time-to MACE (C-Index: 0.6, *p* = <2e-16 across all S_v_). The M_ecg+cac_ model was associated with MACE (C-index: 0.71). Model comparison showed that M_ecg+cac_ was superior to M_ecg_ (*p* = 1.8e-10) or M_cac_ (*p* < 2.2e-16) alone. The M_nom_, comprising of eRiS, CAC, age and sex was associated with MACE (C-index 0.71). eRiS had the most significant contribution, followed by CAC score and other clinical variables. Further, M_nom_ was able to identify unique patient risk-groups based on eRiS, CAC and clinical variables.

**Conclusion:**

The use of ECG features in conjunction with CAC may allow for improved prognostication and identification of populations at risk. Future directions will involve prospective validation of the risk score and the nomogram across diverse populations with a heterogeneity of treatment effects.

## Introduction

Cardiovascular disease is the leading cause of death in the United States, with significant morbidity and cost of care (1). While cardiovascular mortality has declined in recent decades, the rate of decline appears to be decelerating, thought to be related to the increasing prevalence and exposure to risk factors such as unhealthy diet, obesity, physical inactivity, hyperlipidemia, hypertension and high alcohol use (2). Therefore, primary prevention of atherosclerotic cardiovascular disease (ASCVD) remains an important public health goal but requires precise identification of at-risk individuals.

Current approaches for risk evaluation are dependent on probabilistic risk scores, which are poorly calibrated, do not perform well across populations and do not provide individual risk assessment. The Pooled Cohort Equations (PCE) (1), which is a sex- and race-specific tool for estimating 10-year absolute rates of ASCVD is based on nine clinical variables. While PCE is in routine clinical use in the U.S., its CV risk overestimation (3, 4) and suboptimal calibration in specific patient populations have been noted (5–9), leading to updated clinical practice guidelines in 2019 (10, 11). While cardiac death was originally defined in the PCE as only those related to coronary heart disease, other types of cardiac death, such as those related to fatal arrhythmias or heart failure, also occur and may not be captured by the PCE (12–17) Coronary artery calcium (CAC), a marker of atherosclerosis, is an essential predictor of coronary artery disease, incident cardiovascular events and all-cause mortality (18–21). CAC combined with traditional clinical risk factors in an ML model, have been associated with superior risk prediction when compared to PCE and CAC alone (22). Also, combining CAC with traditional risk scores are better predictors of 1-year MACE and early revascularization (23, 24). However CAC may still not capture risk in the context of other indications such as for arrhythmic events.

ECG features have been used to diagnose or predict cardiovascular events. For instance, heterogeneity of R-wave and T-wave morphology and ST-segment elevation have been used to diagnose ventricular arrhythmias (25, 26). ST-segment elevation myocardial infarction (STEMI) has also been used for chronic HF prediction (27). QRS duration and morphology including left bundle branch block have been used for assessing ventricular dyssynchrony and predicting heart failure (28). Similarly, QT-prolongation and T-wave abnormalities are associated with increased risk for arrhythmia (29) and SCD (30), respectively. However, these individual ECG markers require manual assessment and can thus, be prone to subjective interpretation and variable clinical decisions. Recently, machine learning (ML) have been employed to analyze the 12-lead, high-dimensional ECG signal automatically, providing a more quantitative and reproducible alternative to more subjective interpretation (31, 32). Neural networks on ECGs have been shown to outperform manual QTc measurements for life-threatening ventricular arrhythmia prediction (33, 34) and also as predictive tools for ventricular dysfunction (35, 36), coronary artery disease (37), atrial fibrillation (38, 39), myocardial hypertrophy (40) and ischemic heart disease (41). Although ML frameworks on ECGs lack direct interpretability, they have been used to detect the most relevant waves (P-wave, QRS complex or T-wave), contributing to diagnosis of CVDs (42). Additionally, ML frameworks have shown to detect both, clinically significant and other subtle features that are not traditionally used by cardiologists (42).

Given the significant value and success of ML-aided techniques over manual assessment for cardiovascular disease diagnosis and prediction, traditional ASCVD risk calculators can potentially be augmented by features derived from ECG using ML. In addition to ECG and other clinical predictors, inclusion of CAC may improve cardiovascular risk stratification beyond using CAC or ECG alone. This study sought to address three objectives: (1) To evaluate the utility of ML on ECG data (hand-crafted features) to predict MACE, (2) To evaluate the additive benefit of ECG on CAC scores to predict MACE and (3) To construct a nomogram with ECG and clinical variables to assess its predictive capability of MACE. By including ECG and CAC in a single model using a large prospective cohort (5,864 patients), a novel ASCVD-specific risk calculator is presented, one that addresses some of the limitations associated with the PCE.

## Methods

### Study design and participants

We used data from the Community Benefit of No-charge Calcium Score Screening Program (CLARIFY, ClinicalTrials.gov Identifier: NCT04075162), a prospective cohort study of patients with at least one cardiovascular risk factor who underwent no-charge coronary artery calcium scoring at University Hospitals Health Systems (UHHS), comprising 11 hospitals and >31 health centers across Ohio. We included participants with at least 1 cardiovascular risk factor and with a clinically available 12 lead electrocardiogram in CLARIFY who underwent CAC between January 1^st^, 2014, to November 4^th^, 2020. Cardiovascular events were identified from the electronic medical records as part of the registry and included heart failure, myocardial infarction, coronary revascularization, stroke, and death. Available patient factors included CAC, age, female, race, smoking status, body mass index, blood pressure, serum lipids (total cholesterol, low-density lipoprotein, high-density lipoprotein, triglycerides). The 10-year predicted risk of atherosclerotic cardiovascular disease was calculated for individuals with available variables using the AHA/ACC pooled cohort equations.

### ECG feature extraction

During an ECG test, each patient's data is automatically processed through GE Muse™ Cardiology Information System (Milwaukee, WI, USA) using the validated GE Marquette™ 12SL™ ECG analysis program (43). For each 12-lead ECG test, a total of 649 ECG features (53 per lead and 13 overall) were extracted. These features consisted of measurements such as amplitude and interval measurements from all deflections in the ECG waveform in each of the 12 leads (Supplementary Table 1). These 649 features along with cardiovascular event outcomes were extracted for all CLARIFY patients and were made available for subsequent analysis.

### Feature selection and model construction

For this analysis, patients in the CLARIFY trial were split randomly into training (S_tr_) and validation sets (S_v_) using a stratification technique to preserve the same proportion of those who had an adverse CV event vs. those without an event during their follow-up period. Four split sizes were implemented [S_tr_ (1): S_v_ (1): 50:50, S_tr_ (2): S_v_ (2): 60:40, S_tr_ (3): S_v_ (3): 70:30, S_tr_ (4): S_v_ (4): 80:20], and the results were compared across all the subsets.

**Figure 1:**
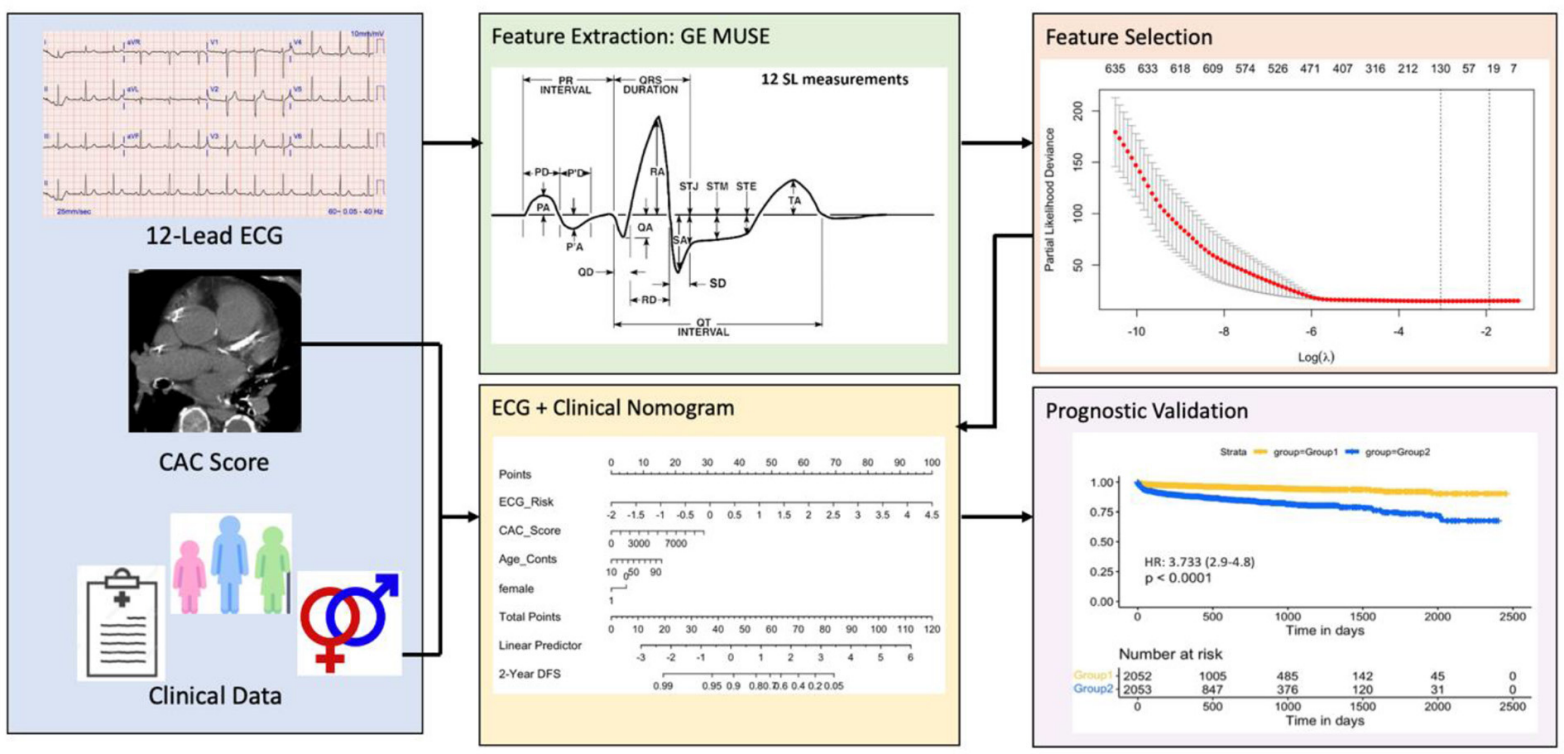
Overall workflow. The first step involves collecting ECG tests, manual CAC scores and clinical data from eligible patients. The ECG features are then automatically extracted using the commercially available GE MUSE software. Top ECG features were selected using the LASSO feature selection method and used for constructing eRiS. M_nom_ was constructed using clinical features and eRiS. M_ecg_ and M_nom_ were validated for prognostic performance and predicting downstream MACE events. LASSO: least absolute shrinkage and selection operator; eRiS: ECG risk score, Mecg: Cox PH model using eRiS alone; Mnom: Nomogram with eRiS, CAC and clinical factors.

The least absolute shrinkage and selection operator (LASSO) (44) method was used to select the most useful predictive features from the patients in S_tr_. The value of the tuning parameter in the LASSO-Cox model (λ) was averaged out *via* 10 cross-validations to minimize error. An advantage of the LASSO based analysis is the sparse solution associated with it, resulting in unimportant features being assigned a weight of 0. As a result, only the most discriminative features are preserved using this strategy. After selecting the top features, the corresponding LASSO coefficients were used for the eRiS construction. eRiS was calculated for each patient *via* a linear combination of selected features that were weighted by their respective coefficients. Hence, for each patient, a new composite ECG-risk score is added as a single feature (Figure 1).

A Cox-proportional hazards model was used to assess the benefit of using an eRiS alone (M_ecg_), CAC alone (M_cac_) and a combination of eRiS and CAC (M_ecg+cac_) for ASCVD that is prognostic of adverse CV disease-free survival. These models were then further validated on S_v_. M_nom_ was constructed by integrating eRiS with CAC and clinical covariates (age and sex) (Figure 1). This was developed on S_tr_ and then validated on S_v_. To validate M_nom_ against the standard PCE based risk calculator (1), we extracted the estimated 10-year ASCVD risk from PCE for patients in this study. This information was available for only 1,291 patients out of the 5,864 eligible patients analyzed in this study.

### Outcomes

The primary endpoint of the study was the prognostic performance of M_ecg_, M_cac_, M_ecg+cac_ and M_nom_ with respect to CV risk prediction, which was measured from the date of CAC scoring to the time of composite cardiovascular event. Patients who were alive and did not have an event were censored at the date of last follow-up. We validated whether performance of M_nom_ was statistically better when compared to M_ecg+cac_ and M_ecg_. A second objective of our study was to assess whether eRiS in addition to CAC score (M_ecg+cac_) could provide additional benefit to CAC when predicting CV risk. Other objectives of our study were to assess whether M_ecg_ and M_nom_ could be used to identify patient cohorts at higher probability for developing major adverse cardiovascular events (MACE: defined as composite of myocardial infarction, coronary revascularization, stroke, heart failure, or death).

### Statistical analysis

The risk determination of M_ecg_ and M_cac_ was validated using hazard ratios (HR) (95% CI) and Harrell's concordance index. Further, the fit for the combination model M_ecg+cac_ was evaluated against M_ecg_ and M_cac_ using ANOVA. The benefit of addition of eRiS to CAC in CV risk prediction was evaluated using C-indices and HRs of eRiS and CAC. For prognostic stratification, eRiS values were used to divide the training cohort into two groups for which MACE-free survival and HRs were calculated. The prognostic performance of M_ecg_ was validated using Kaplan-Meier survival analysis, log-rank test, HR (95% CI), and Harrell's concordance index [C index (95% CI)]. Univariate analysis of eRiS and the clinical variables was performed. Multivariable Cox-regression analysis was used to investigate the relationships between the various covariates and 2-year MACE-free survival. To assess nomogram risk discrimination, C indices were calculated from the nomogram for eRiS alone, CAC alone and clinical risk factors alone.

## Results

### Patient population

A total of 5,864 eligible patients were included in this study. Baseline characteristics are listed in Table 1. Over a median follow-up of 14.3 months, 73 died (1.2%), 220 had heart failure (HF) (3.8%), 71 had myocardial infarction (MI) (1.2%), 104 had stroke (1.8%), 235 had MACE (Death/MI/Stroke), 106 had Revascularization (coronary artery bypass graft surgery/percutaneous coronary intervention) (1.8%), and 494 had composite MACE (8.4%). CAC score distribution was skewed to the right with 37% patients with CAC score 0, and mean score 250 [0–9,479].

**Table 1:** Patient baseline characteristics.

**Variables**	
Calcium score	19.5 [0.0–217.0] (*n* = 5,864)^*^
Age	60.0 [53.0–67.0] (*n* = 5,864)^*^
Gender	Female: 53% (*n* = 3,091) Male: 47% (*n* = 2,773)
Race	White: 83% (*n* = 4,854) Black: 14% (*n* = 834) Other: 1.6% (*n* = 92) Unknown: 1.4% (*n* = 84)
Smoking status	Non-Smokers: 61% (*n* = 3,592) Smokers: 39% (*n* = 2,272)
Body mass index (kg/m^2^)	29.57 [25.9–34.2] (*n* = 5,533)^*^ Unknown: 6% (*n* = 331)
Systolic blood pressure (mmHg)	130 [120–142] (*n* = 5,567)^*^ Unknown: 5% (*n* = 297)
Diastolic blood pressure (mmHg)	80 [72–84] (*n* = 5,568)^*^ Unknown: 5% (*n* = 296)
High-density lipoprotein cholesterol (HDL-C), mg/dl	50 [41–61.5] (*n* = 3,772)^*^ Unknown: 36% (*n* = 2,092)
Low-density lipoprotein cholesterol (LDL-C), mg/dl	113 [88–140] (*n* = 3768)^*^ Unknown: 36% (*n* = 2,096)
Total cholesterol, mg/dl	193 [163–223] (*n* = 3,846)^*^ Unknown: 34% (*n* = 2,018)
Triglycerides, mg/dl	114 [81–165] (*n* = 3,744)^*^ Unknown: 36% (*n* = 2,120)

* Median [IQR].

### ECG-based risk score construction

The LASSO-Cox regularization model resulted in preservation of 89, 119, 89, and 115 features, respectively, with 27 features being common to all across S_tr_ (1), S_tr_ (2), S_tr_ (3) and S_tr_ (4). The selected features are listed in Supplementary Table 2. This feature selection methodology preserved only about 18% of the initial extracted features, representing the highly correlated nature of the signal itself.

### Cox proportional model analysis

#### ECG-risk score alone model (M_ecg_)

**Figure 2:**
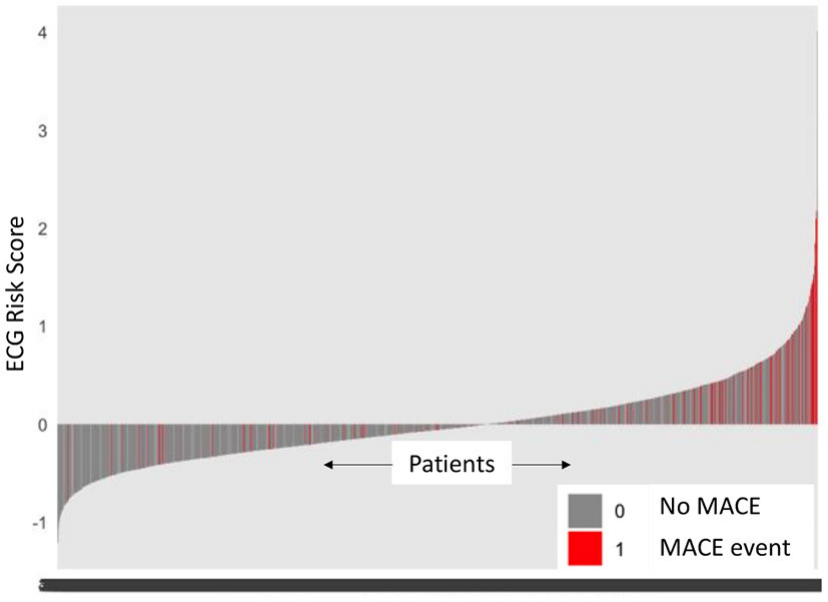
ECG risk score predicts MACE events. Patients with higher ECG risk score (eRiS) correlate with occurrence of MACE events, demonstrating the value of considering ECG as a factor in determining probability of a MACE event. X-axis denotes patients arranged in ascending value of eRiS scores.

A Cox proportional model (M_ecg_) comprising of eRiS alone predicted time-to-MACE across all data splits (C-Index: 0.6, p = <2e-16 across all S_v_): S_v_ (1) [HR: 2.98 (2.3–3.87)], S_v_ (2) [HR: 2.26 (1.77–2.9)], S_v_ (3) [HR: 5.09 (4.14–6.25)], S_v_ (4) [HR: 2.74 (2.04–3.67)]. Association of downstream MACE events to eRiS was visualized in Figure 2. It was observed that patients with higher eRiS score tended to have a higher probability of a MACE event.

**Figure 3:**
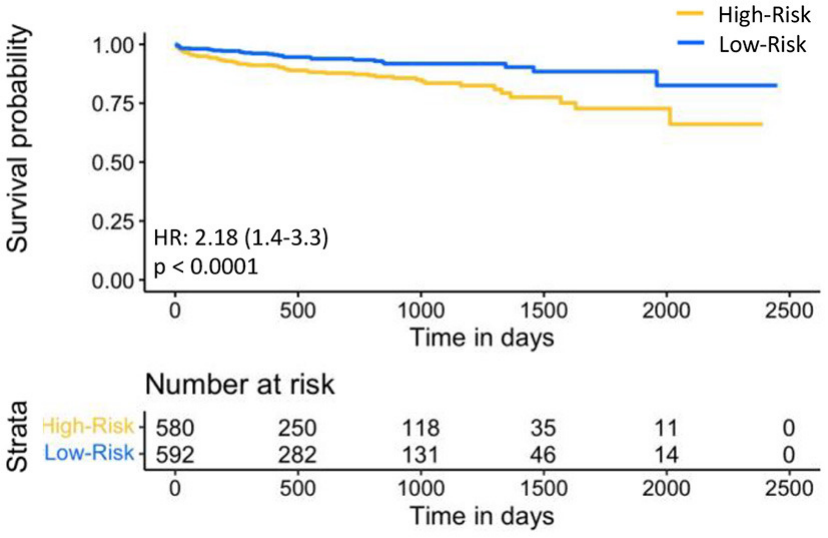
Kaplan-Meier plot for MACE-free survival according to eRiS-based risk groups in S_v_. The eRiS threshold of −0.055 showed two distinct groups of high vs. low MACE-free survival in S_v_ (4).

For prognostic validation, two groups were identified in S_tr_ using eRiS median threshold. For instance, in S_tr_ (4), threshold was −0.055, below which patients were observed to have low risk of MACE (and hence increased MACE-free survival) and above which patients were observed to have high risk of MACE (hence decreased MACE-free survival). Kaplan-Meier for MACE-free survival were plotted to visualize patient MACE survival over follow-up time in our validation group (Figure 3).

#### CAC alone model (M_cac_)

M_cac_ was found to be significantly associated with CV events. (C-index 0.7, *p* = <2e-16).

#### ERiS ± CAC model (M_ecg±cac_)

**Figure 4:**
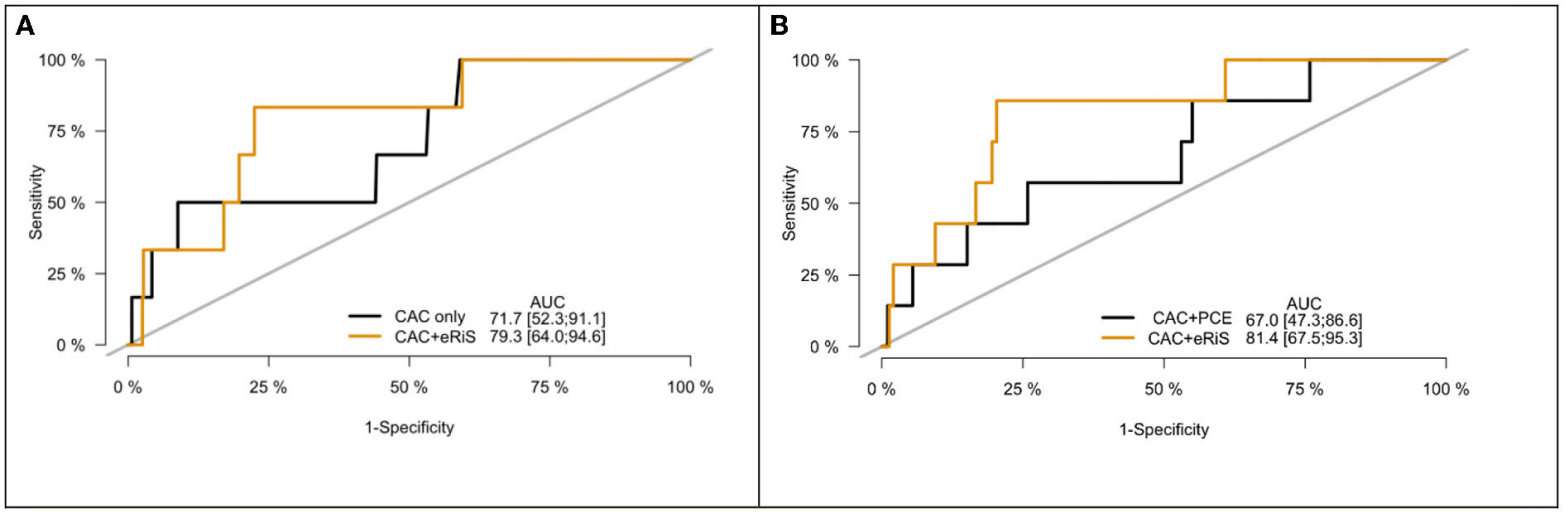
Receiver operating characteristic (ROC) curve for CAC only vs. eRiS+CAC shows the benefit of adding eRiS to CAC for better prediction of the probability of a MACE event in **(A)**. CAC+eRiS showed better performance than CAC+PCE (C-index: 0.72 vs. 0.67) for patients who had PCE available in **(B)**.

**Figure 5:**
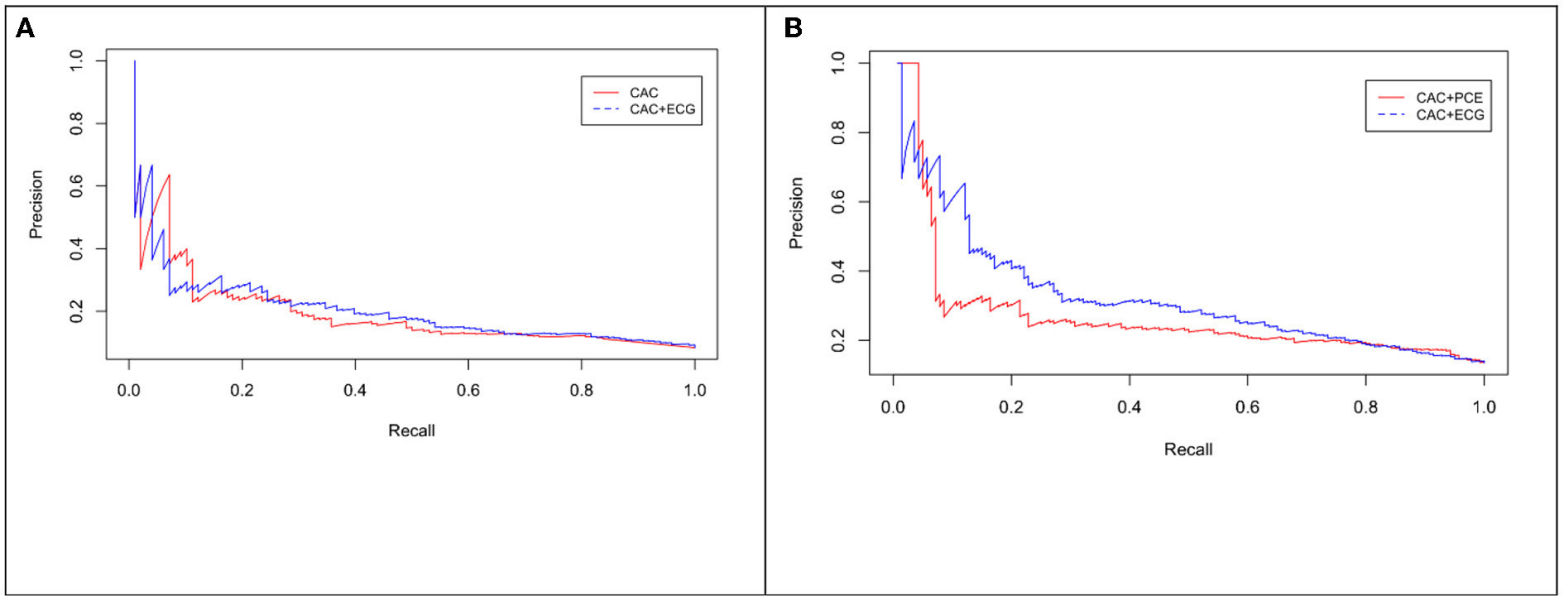
Precision-Recall (PR) curve for CAC only vs. eRiS+CAC shows the benefit of adding eRiS to CAC for better prediction of the probability of a MACE event in **(A)** (Average F1 statistic 0.20 vs. 0.21. PR AUC: 0.68 vs. 0.71). **(B)** CAC+eRiS showed better performance than CAC+PCE for patients who had PCE available (Average F1 statistic 0.28 vs. 0.30. PR AUC: 0.68 vs. 0.71).

**Figure 6:**
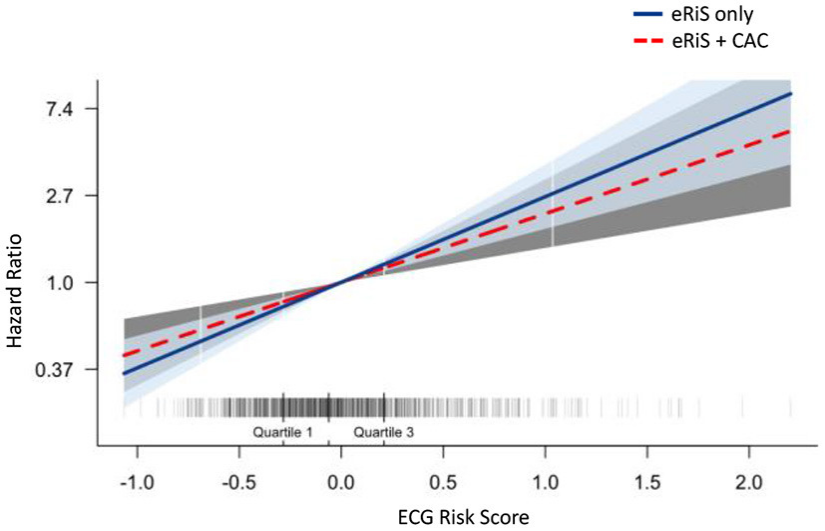
Additive benefit of eRiS to CAC: Hazard ratio is not attenuated when eRiS is adjusted by CAC score, indicating a strong relationship with MACE which is not weakened by the addition of CAC. Similar results were seen with other splits.

M_ecg+cac_ was significantly associated with cardiovascular events (C-index: 0.65, *p* = <2e-16). Model comparison using ANOVA showed that M_ecg+cac_ performed in a manner that was statistically superior when compared to M_cac_ (*p* < 2.2e-16) (Figures 4A, 5A). M _ecg+cac_ also performed better in comparison to M_CAC+PCE_ (*p* < 2.2e-16) (Figures 4B, 5B). Additionally, the adjustment of CAC to eRiS score did not attenuate HR for eRiS. Figure 6 shows this for S_v_ (4). Similar results were seen for other splits.

**Figure 7:**
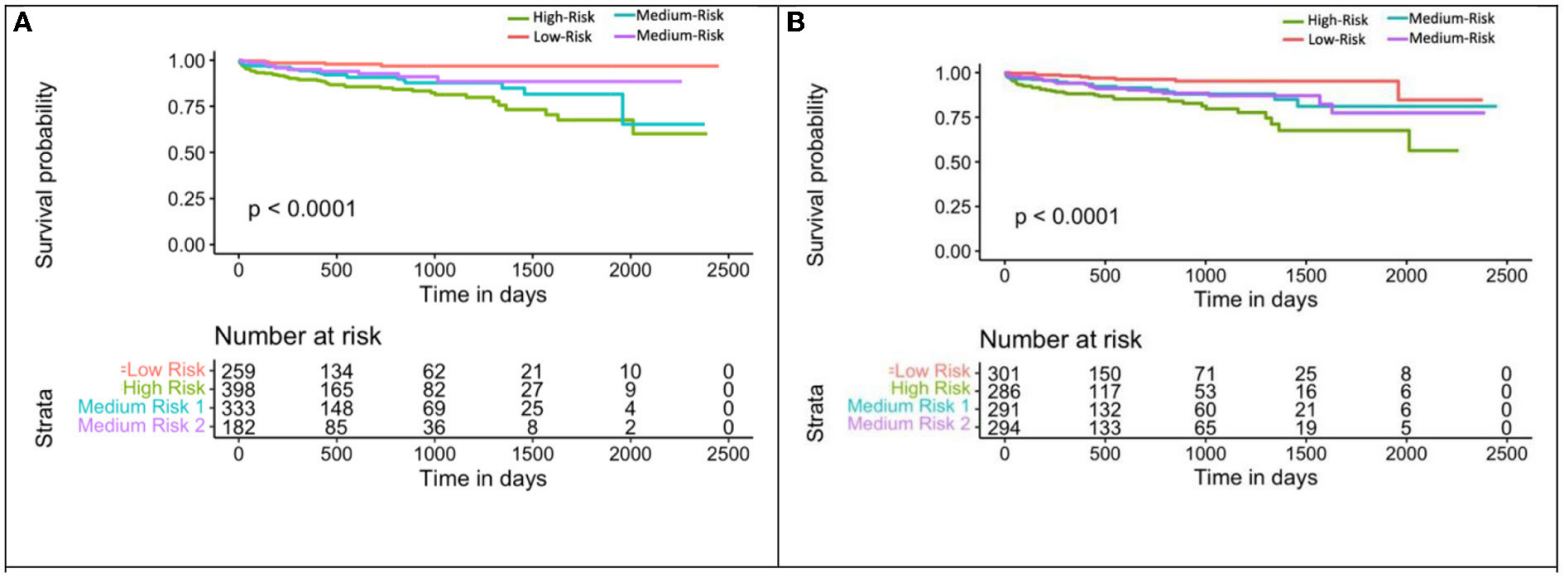
Kaplan-Meier plot for MACE-free survival according to eRiS+CAC and eRiS only risk groups in S_v_ (4). **(A)** The eRiS+CAC threshold showed worse prognosis for patients with high eRiS combined with high CAC score. HR between high and low risk: 6.72 [4.42–10.22]. **(B)** Similar observation seen in eRiS based segregation. HR between high and low risk: 5.22 [2.54–10.75].

M_ecg+cac_ was used to divide the population into 4 groups: (1) eRiS<median and CAC=0; (2) eRiS<median and CAC>0; (3) eRiS> median and CAC=0; (4) eRiS> median and CAC>0 (Figures 6, 7). As seen in the figure, worst prognosis was seen in the group with higher eRiS and CAC score, showing the additive benefit of ECG to CAC for MACE survival prediction. Similar groupings were seen when eRiS alone was used to divide S_tr_ into 4 groups, although higher HRs were observed between high and low risk patients with the eRiS+CAC model. These observations were validation on the held-out validation datasets (S_v_).

We also evaluated reclassification index into high vs. low risk based of eRiS and CAC scores. We considered a threshold of −0.055 (eRiS median threshold in training data) for ECG and 400 for CAC for this analysis. In S_v_ (4), 41% of patients with CAC score of 0 were reclassified as high risk by ECG. Further, 38% of patients with CAC>400 were reclassified as low risk by ECG.

#### ECG, CAC and clinical factors nomogram (M_nom_)

**Figure 8:**
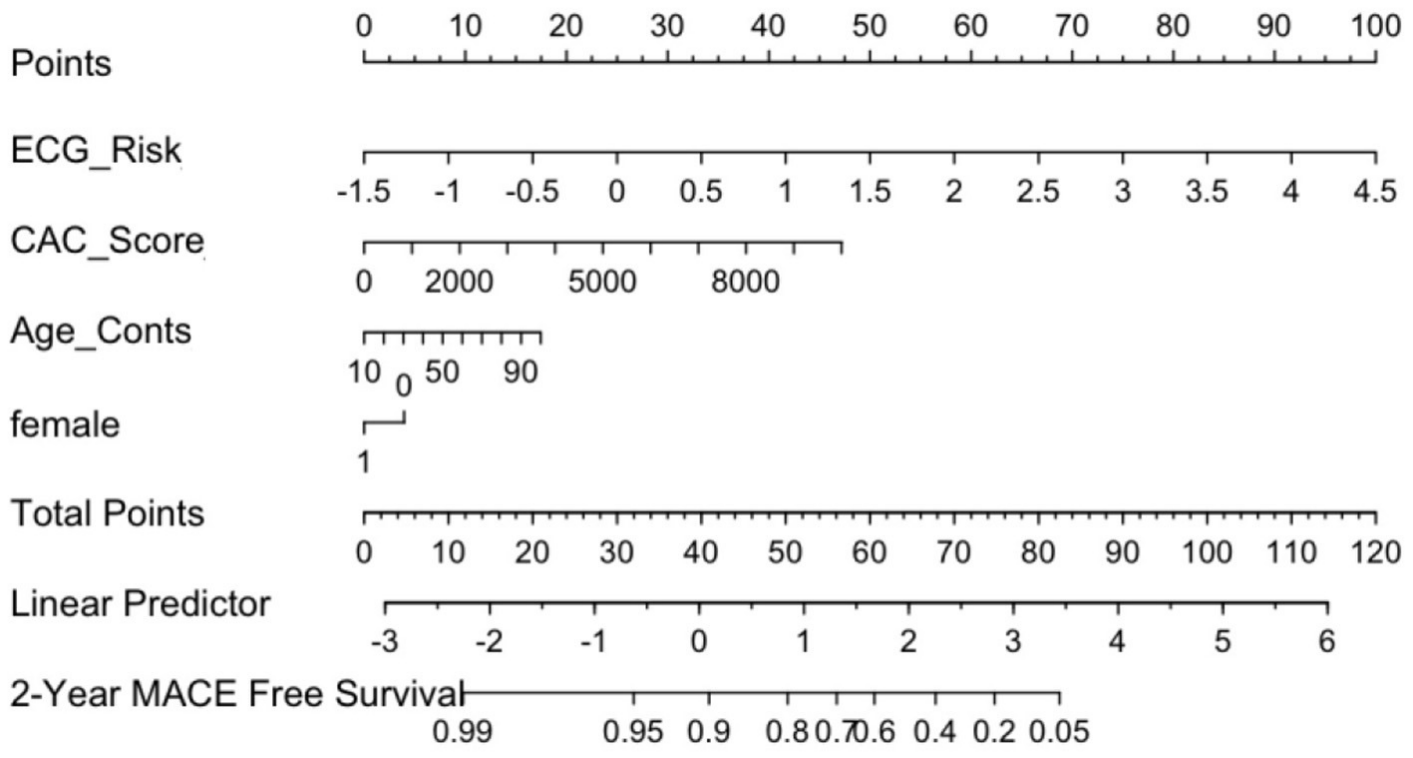
ECG Nomogram (M_nom_) demonstrates relative contribution of each covariate in MACE prediction. ECG risk score has the most significant contribution, followed by CAC score and other clinical variables.

The calibration curve for the nomogram showed agreement between predicted survival and actual survival, and the C index for M_nom_ was 0.76. As shown in Figure 8, the ECG risk score had the most significant contribution, followed by CAC score and other clinical variables.

**Figure 9:**
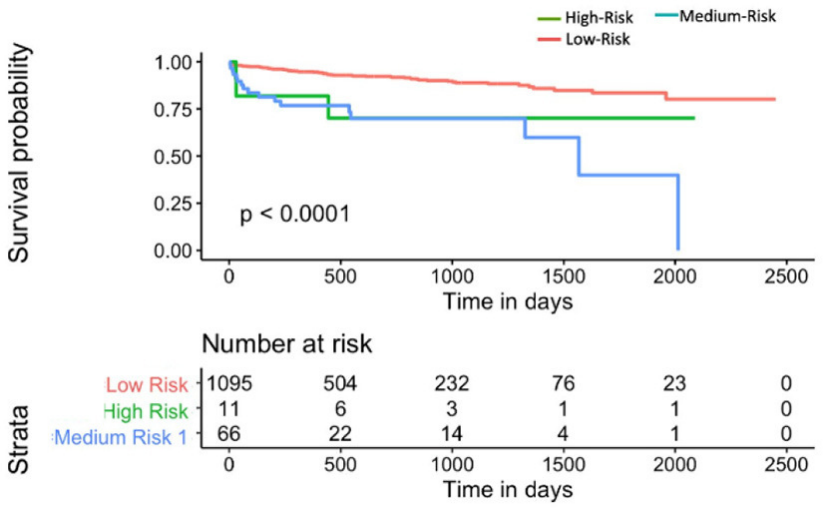
Kaplan-Meier plot for MACE-free survival according to M_nom_ risk groups for S_v_ (4) [C-index 0.6 (se = 0.023]). HR between high and low risk: 3.24 [1.02–10.30].

M_nom_ was also used to divide the population into high, medium and low risk groups using thresholds of 20 and 60% MACE free survival. As illustrated in Figure 9, M_nom_ predictions resulted in high and low risk groups. The two groups were significantly different in terms of their survival

#### ECG risk score-based nomogram vs. PCE for MACE survival prediction

M_nom_ was found to be statistically superior in terms of prognosis, to M_PCE_ across all Sv (C-index: 0.71 vs. 0.68; *p* < 0.001).

## Discussion

Current ACC/AHA guidelines for atherosclerotic cardiovascular disease (ASCVD) risk assessment involves incorporation of traditional risk factors but does not include additional dimensions of risk that may be conveyed through other modalities such as coronary artery calcium (CAC) and electrocardiography (ECG). Improving prediction of major adverse cardiovascular events (MACE) risk can help identify at risk patients who may benefit from treatment interventions. Conversely, identification of patients who are at low risk of MACE, might help prevent the potentially harmful impact of unnecessary treatments. In this study, we developed and validated a novel ECG and CAC-based nomogram, that was not only associated with likelihood of MACE but, improved cardiovascular risk stratification when compared to the frequently used PCE for ASCV risk estimation calculator.

CT-based coronary artery calcium scoring has shown to be the single best predictor of CHD and CVD and is currently endorsed by clinical practice guidelines in select populations (21, 45, 46). CAC scoring, however, has modest discrimination for total CVD events (e.g., inclusive of heart failure event and arrhythmic events), which is becoming an important composite outcome. Additionally, CAC does not involve electrophysiologic parameters present in ECG (e.g., QRS width, q-waves, AV block) that can be markers of CV risk. Therefore, one of the main objectives of this study was to combine both electrophysiological and CT-based diagnostic information to improve CV risk prediction and prognosis when compared to either using ECG or CAC alone.

Previous studies have assessed associations of ECG with CAC in certain cardiac conditions. For instance, one study noted CAC scores being higher with ECG abnormalities as compared to those with normal ECGs and an elevated CAC burden with myocardial disease (47). In another study, QT interval duration significantly correlated with CAC in diabetic patients (48). Further, presence of both CAC and abnormal ECG has been associated with the highest rate of coronary events (49). Our study differs from these works in that we employed ML on a standard 12-lead ECG to automatically select relevant features and use these features to construct a risk score which was then tested for additive prognostic ability with CAC. Our ECG-CAC model was significantly associated with cardiovascular events in our dataset comprising of 5,864 patients. Additionally, our ECG-CAC model performed in a manner that was statistically superior compared to ECG or CAC alone or CAC-PCE, thus demonstrating the value of combining ECG and CAC for MACE prediction. We also demonstrated the risk reclassification by incorporating both ECG and CAC into MACE prediction. For patients with CAC score of 0, a higher risk predicted by ECG features may help guide clinical decision-making toward statin prescription recommendations.

A dedicated nomogram was developed demonstrating the relative contribution of ECG, CAC with traditional clinical factors for CV risk prediction. As seen in the nomogram, ECG risk score, which is currently not included in routine clinical practice for ASCVD risk prediction, was the most significant contributor to risk prediction. When tested against PCE for patients with this information, our nomogram performed at a level that was statistically superior to PCE. This suggests that incorporation of anatomic imaging (CAC), physiologic data (ECG) and clinical variables is superior to employing each stream of data independently. Our nomogram was also used to assess high vs. low risk patients based on their ECG risk scores and clinical variables. Patients in the low-risk group had higher survival rates compared to the high-risk patients. These findings appear to suggest that our novel tool, when deployed in the clinic, could be used for triaging patients far superior to PCE. ECG signals are routinely used as part of CAC imaging for image gating and thus are routinely available. A fully automated platform incorporating ECG, CAC and clinical variables can be envisioned for accurate risk prediction.

Much prior work around developing an ECG risk score has been based on using specific leads or ECG waveforms to predict CV risk/events (50). One study, for instance, used P-wave variables to stratify patients into 3 risk groups (51). Manually annotated features from ECG reports have been used to calculate an ECG risk score, associated with sudden cardiac death and risk stratification (52). ECG risk equation based on age, sex, QT interval, heart rate, and T axis was shown to be comparable to the Framingham risk score and yielded significant improvement in risk classification (53). Although ECG based risk scores have been proposed in the past, most of these were not employed in a way to take advantage of the entire 12-lead signal. Additionally, to our knowledge, no study has tested the impact of ECG-based risk score in conjunction with CAC scoring to predict MACE.

Due to the high-dimensional electrophysiological information captured in a single 12-lead ECG test, previous studies have utilized sophisticated ML techniques, including deep learning (DL) to automatically diagnose and predict various cardiovascular outcomes (54). For instance, convolutional neural networks (CNN) on ECGs have identified patients with atrial fibrillation with high accuracy (39). DL models have also been developed to automatically interpret ECG abnormality types (55). Similarly, DL algorithms on routine 12-lead ECGs have been used to detect low ejection fraction (36), arrhythmia detection (56), aortic stenosis (57), atrial fibrillation (58), heart failure (59) and even all-cause mortality (60). Additionally, wavelets have also been used to extract features from ECGs for LV diastolic dysfunction detection using ML models (61). Also, unsupervised ML of ECG have been used to stratify which CRT candidates may have better response to resynchronization therapy beyond using QRS duration and left bundle branch block (62). However, all prior studies have relied upon DL methodologies employing neural networks or ML models. While these approaches are powerful and do not require domain level expertise to employ, the abstract nature of DL transformations often preclude any clear, clinically meaningful explanation of the features that drive the model predictions.

Recognizing that adoption of automated signal analysis platforms into clinical practice will require not only convincing statistical demonstrations but also clear, transparent, and biologically inspired methods, we chose to employ a “hand-crafted” feature-engineering approach in this work. This approach relied on using clinically validated ECG features automatically extracted from the GE Muse™ Cardiology Information System. Our risk score comprised of ECG features selected from a standard 12-lead ECG using ML to preserve the most important features. A major advantage of our score is the fact that no manual annotation is required to define the ECG features. As seen in this study, the ML derived ECG risk score was able to improve stratification of high-risk ASCVD patients, thus potentially helping physicians with identification of such patients. Another strength of this study was the relatively large sample size, and the inclusion of the CAC score, which is arguably considered the best marker of MACE risk.

This study did have its limitations. First, the ECG features used here were those that were automatically extracted from the GE MUSE system. Although this widely available clinical software has been validated with manual annotations, disadvantages might include inability to capture other subtle features in the ECG waveform beyond what is offered from the system. Second, the patient datasets used in this study consisted only of those who were part of the UH health system and are subject to referral bias. Although ECG, ECG-CAC and the subsequent nomogram showed consistent performance with respect to predicting patients with MACE, this tool needs to be validated prospectively in an external cohort with a diverse population in a multi-institution setting. In this current study, we were unable to compare to the MESA 10 -year CHD risk with CAC model, which may be current standard of care for many clinicians when making decisions from CAC scores. This will be considered in future analyses. Third, effects of treatment, if any, has not been considered in this study. Future studies could be directed toward adding more treatment-related variables into the nomogram, thus capturing the heterogeneity in the ASCVD prediction effects. As shown in this study, CAC score along with the ECG provided an additive benefit in ASCVD risk prediction. This suggests that assessing features from the CAC score itself such as CT radiomics features (63) with ECG omics, might provide even better risk stratification.

## Conclusion

We developed and validated an ECG risk score-based model, ECG incorporated with CAC and a novel nomogram, with ECG, CAC and clinical factors. These models were implemented on various training-validation dataset sizes and the ECG features extracted were overall consistent. The nomogram identified high vs. low risk patients for downstream MACE with high separability. Following prospective multi-site validation, the ECG score could be incorporated in the electronic medical systems of patients which would enable personalization of treatment regimens with the addition of ECG based information. Specifically, it could be employed as a clinical decision tool to enable triaging patients based on ECG, CAC and the patient-specific clinical factors. Future testing is needed to evaluate how clinical CVD outcomes may be affected by incorporating this risk estimation tool into primary prevention efforts.

## Data availability statement

The raw data supporting the conclusions of this article can be made available by the authors upon IRB approval and data sharing agreement.

## Ethics statement

The studies involving human participants were reviewed and approved by UH Institutional Review Board with waiver of consent [Community Benefit of No-charge Calcium Score Screening Program-CLARIFY (NCT04075162)].

## Author contributions

SS and SA-K: contributed equally toward data analysis, model building, and manuscript writing. SA-K, NT, and VR: access and acquisition of data. PF: statistical expertise. SR and AM: guidance on entire study, from data collection to data analysis, and manuscript writing. All authors contributed to the article and approved the submitted version.

## Funding

Research reported in this publication was supported by the National Cancer Institute under (award numbers R01CA268287A1, U01CA269181, R01CA26820701A1, R01CA249992-01A1, R01CA202752-01A1, R01CA208236-01A1, R01CA216579-01A1, R01CA220581-01A1, R01CA257612-01A1, 1U01CA239055-01, 1U01CA248226-01, and 1U54CA254566-01), National Heart, Lung and Blood Institute (1R01HL15127701A1 and R01HL15807101A1), National Institute of Biomedical Imaging and Bioengineering 1R43EB028736-01, National Center for Research Resources under award number 1 C06 RR12463-01, VA Merit Review Award IBX004121A from the United States Department of Veterans Affairs Biomedical Laboratory Research and Development Service the Office of the Assistant Secretary of Defense for Health Affairs, through the Breast Cancer Research Program (W81XWH-19-1-0668), the Prostate Cancer Research Program (W81XWH-20-1-0851), the Lung Cancer Research Program (W81XWH-18-1-0440, W81XWH-20-1-0595), the Peer Reviewed Cancer Research Program (W81XWH-18-1-0404, W81XWH-21-1-0345, W81XWH-21-1-0160), the Kidney Precision Medicine Project (KPMP) Glue Grant, and sponsored research agreements from Bristol Myers-Squibb, Boehringer-Ingelheim, Eli-Lilly and Astrazeneca. The funder was not involved in the study design, collection, analysis, interpretation of data, the writing of this article, or the decision to submit it for publication.

## Conflict of interest

AM is an equity holder in Picture Health, Elucid Bioimaging, and Inspirata Inc. Currently he serves on the advisory board of Picture Health, Aiforia Inc, and SimBioSys. He also currently consults for Biohme, SimBioSys and Castle Biosciences. He also has sponsored research agreements with AstraZeneca, Boehringer-Ingelheim, Eli-Lilly and Bristol Myers-Squibb. His technology has been licensed to Picture Health and Elucid Bioimaging. He is also involved in 3 different R01 grants with Inspirata Inc. The remaining authors declare that the research was conducted in the absence of any commercial or financial relationships that could be construed as a potential conflict of interest.

## Publisher's note

All claims expressed in this article are solely those of the authors and do not necessarily represent those of their affiliated organizations, or those of the publisher, the editors and the reviewers. Any product that may be evaluated in this article, or claim that may be made by its manufacturer, is not guaranteed or endorsed by the publisher.

## Author disclaimer

The content is solely the responsibility of the authors and does not necessarily represent the official views of the National Institutes of Health, the U.S. Department of Veterans Affairs, the Department of Defense, or the United States Government.
