# Supplementary material for: Machine learning derived ECG risk score improves cardiovascular risk assessment in conjunction with coronary artery calcium scoring
Source: Front Cardiovasc Med. 2022 Oct 5;9:976769. doi: 10.3389/fcvm.2022.976769 (PMC9580025; doi:10.3389/fcvm.2022.976769)
Supplement: Supplementary file 1 [file Table_1.pdf]

Supplementary

Table 1: ECG Feature Extraction:

For each 12-lead ECG test, a total of 649 ECG features (53 per lead and 13 overall) were automatically extracted using commercially available software (GE Muse™ Cardiology Information System, GE Healthcare). The features consisted of measurements such as duration, amplitude, and signal intensity, characterizing signal output from each of the 12-leads.

ECG Feature Parameters	
**Overall Features**	P_Onset, P_Offset, QRS_Onset, QRS_Offset, T_Onset, T_Offset, NumQRSComplexes, QRS_Duration, QT_Interval, QTc_Bazett, PR_Interval, VentRate, AvgRRInterval
**Features per 12-lead** (I, II, III, aVL, aVF, aVR, V1, V2, V3, V4, V5, V6)	P_OnsetAmpl, P_PeakAmpl, P_Duration, P_Area, P_PeakTime, PP_PeakAmpl, PP_Duration, PP_Area, PP_PeakTime, Q_PeakAmpl, Q_Duration, Q_Area, Q_PeakTime, R_PeakAmpl, R_Duration, R_Area, R_PeakTime, S_PeakAmpl, S_Duration, S_Area, S_PeakTime, RP_PeakAmpl, RP_Duration, RP_Area, RP_PeakTime, SP_PeakAmpl, SP_Duration, SP_Area, SP_PeakTime, STJ, STM, STE, MaxST, MinST, T_Special, QRS_Balance, QRS_Deflection, Max_R_Ampl, Max_S_Ampl, T_PeakAmpl, T_Duration, T_Area, T_PeakTime, TP_PeakAmpl, TP_Duration, TP_Area, TP_PeakTime, T_End, PFull_Area, QRS_Area, TFull_Area, QRSint, BitFlag
