# Supplementary material for: Machine learning derived ECG risk score improves cardiovascular risk assessment in conjunction with coronary artery calcium scoring
Source: Front Cardiovasc Med. 2022 Oct 5;9:976769. doi: 10.3389/fcvm.2022.976769 (PMC9580025; doi:10.3389/fcvm.2022.976769)
Supplement: Supplementary file 2 [file Table_2.pdf]

Supplementary

Table 2: ECG Feature Selection:
Using LASSO-Cox analysis, top features were selected and used to construct an ECG Risk Score (eRiS). This was done across the different training-validation splits. 28 features were consistently picked by the 4 split sizes. The consensus of features across the splits is listed in this table

**ECG Feature Parameters: Consensus**		
**Overall**		NumQRSComplexes, PR_Interval, P_Offset, P_Onset, QTc_Bazett, VentRate
**Leads**	**I**	QRSint, RP_Area
	**II**	TFull_Area, TP_PeakAmpl
	**III**	T_Duration
	**aVL**	-
	**aVF**	-
	**aVR**	P_PeakAmpl, QRS_Deflection, TFull_Area, T_Special
	**V1**	PP_Area, T_PeakAmpl, T_Special
	**V2**	T_PeakTime
	**V3**	Max_S_Ampl, PP_Duration, QRS_Deflection, T_End
	**V4**	-
	**V5**	MaxST,
	**V6**	RP_PeakTime, TFull_Area, TP_PeakTime, T_Area
